# Supplementary material for: Detergent modulates the conformational equilibrium of SARS-CoV-2 Spike during cryo-EM structural determination
Source: Nat Commun. 2023 May 3;14:2527. doi: 10.1038/s41467-023-38251-9 (PMC10154187; doi:10.1038/s41467-023-38251-9)
Supplement: Supplementary file 1 — Supplementary Information [file 41467_2023_38251_MOESM1_ESM.pdf]

SUPPLEMENTARY FIGURES

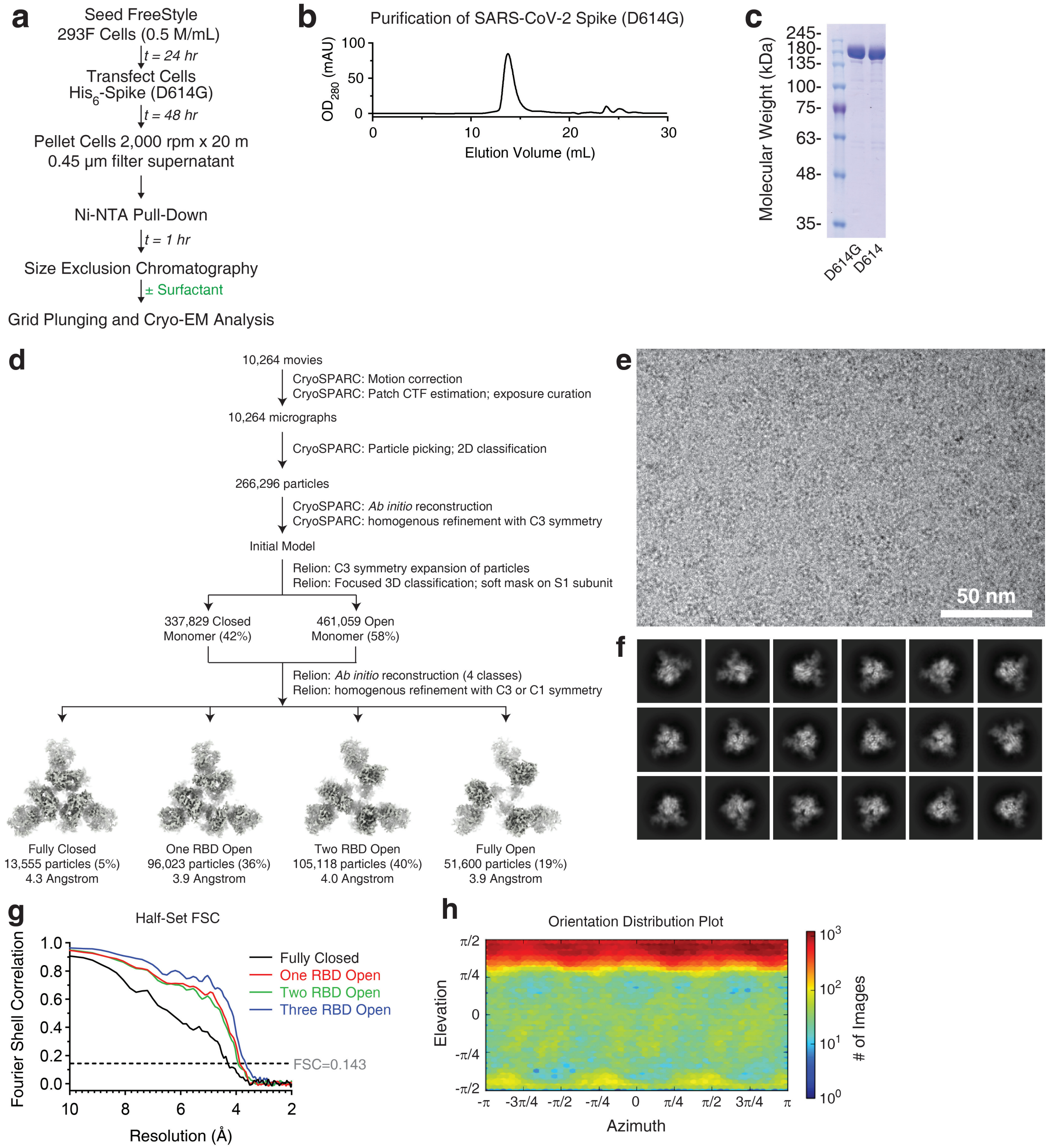

Supplementary Figure 1. **Structural determination of the SARS-CoV-2 D614G Spike at physiological pH (pH 7.4) in the absence of detergent. (a)** Scheme for purification strategy of SARS-CoV-2 Spike protein. **(b)** Gel filtration profile for the SARS-CoV-2 D614G Spike protein at physiological pH (pH 7.4). **(c)** Coomassie staining gel for the eluate following size exclusion chromatography. SARS-CoV-2 Spike protomer can be visualized. Three independent gels were analyzed and a representative is shown. Source data are provided as a Source Data file. **(d)** Workflow for the data processing of the D614G Spike in the absence of detergent. **(e)** A representative raw cryo-EM micrograph for this dataset is shown. In total, 10,264 micrographs were

collected and analyzed for this dataset. **(f)** 2D clustering of extracted particles reveals identifiable features. **(g)** Half-set gold-standard Fourier shell correlation (FCS) for the four classes of Spike conformations identified in this dataset. **(h)** Orientation distribution plot for the D614G Spike in the absence of detergent.

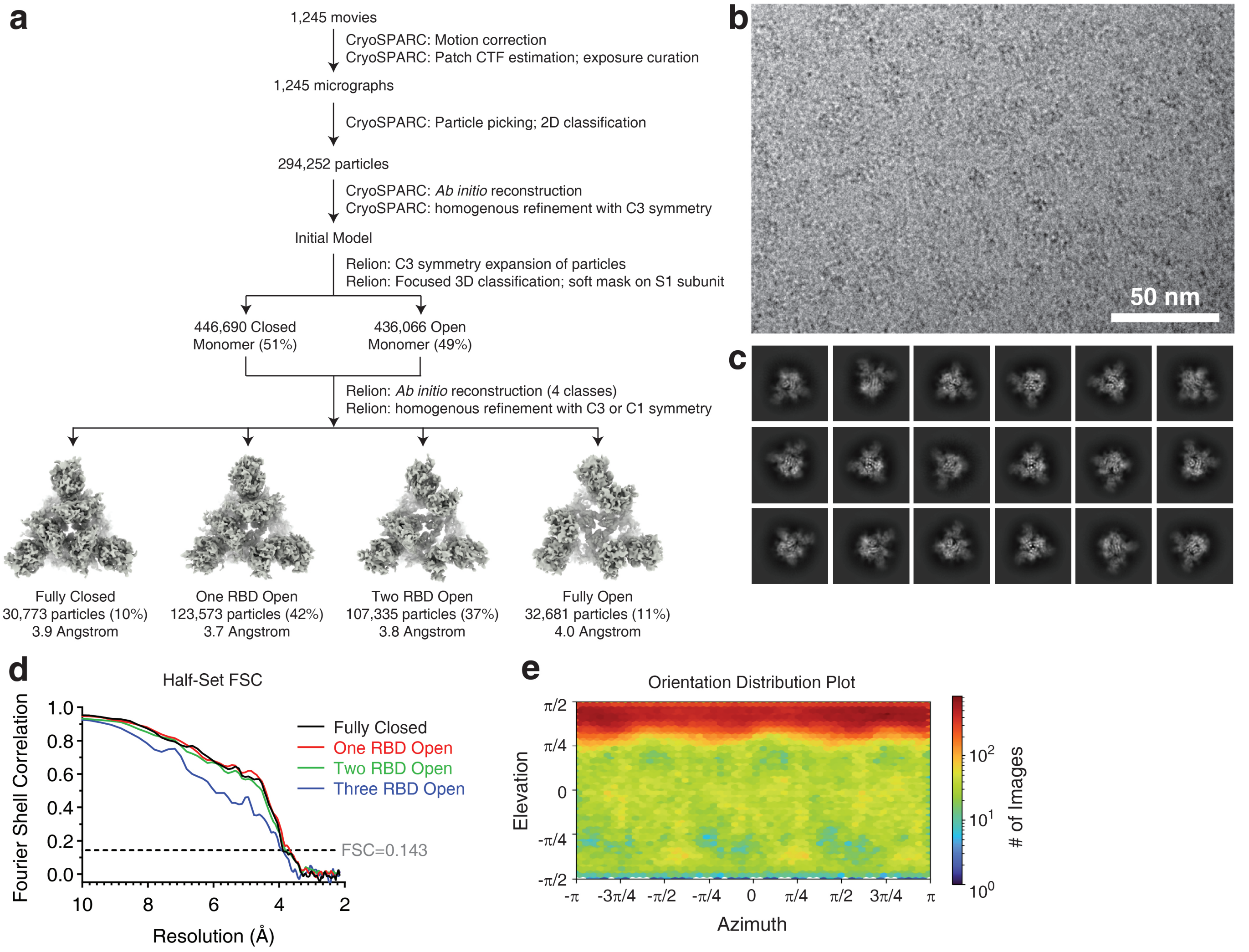

Supplementary Figure 2. Structural determination of the SARS-CoV-2 D614G Spike at physiological pH (pH 7.4) in the presence of 0.01% CHAPS. (a) Workflow for the data processing of the D614G Spike in the presence of 0.01% CHAPS. **(b)** A representative raw cryo-EM micrograph for this dataset is shown. In total, 1,245 micrographs were collected and analyzed for this dataset. **(c)** 2D clustering of extracted particles reveals identifiable features. **(d)** Half-set gold-standard Fourier shell correlation (FCS) for the four classes of Spike conformations identified in this dataset. **(e)** Orientation distribution plot for the D614G Spike in the presence of 0.01% CHAPS.

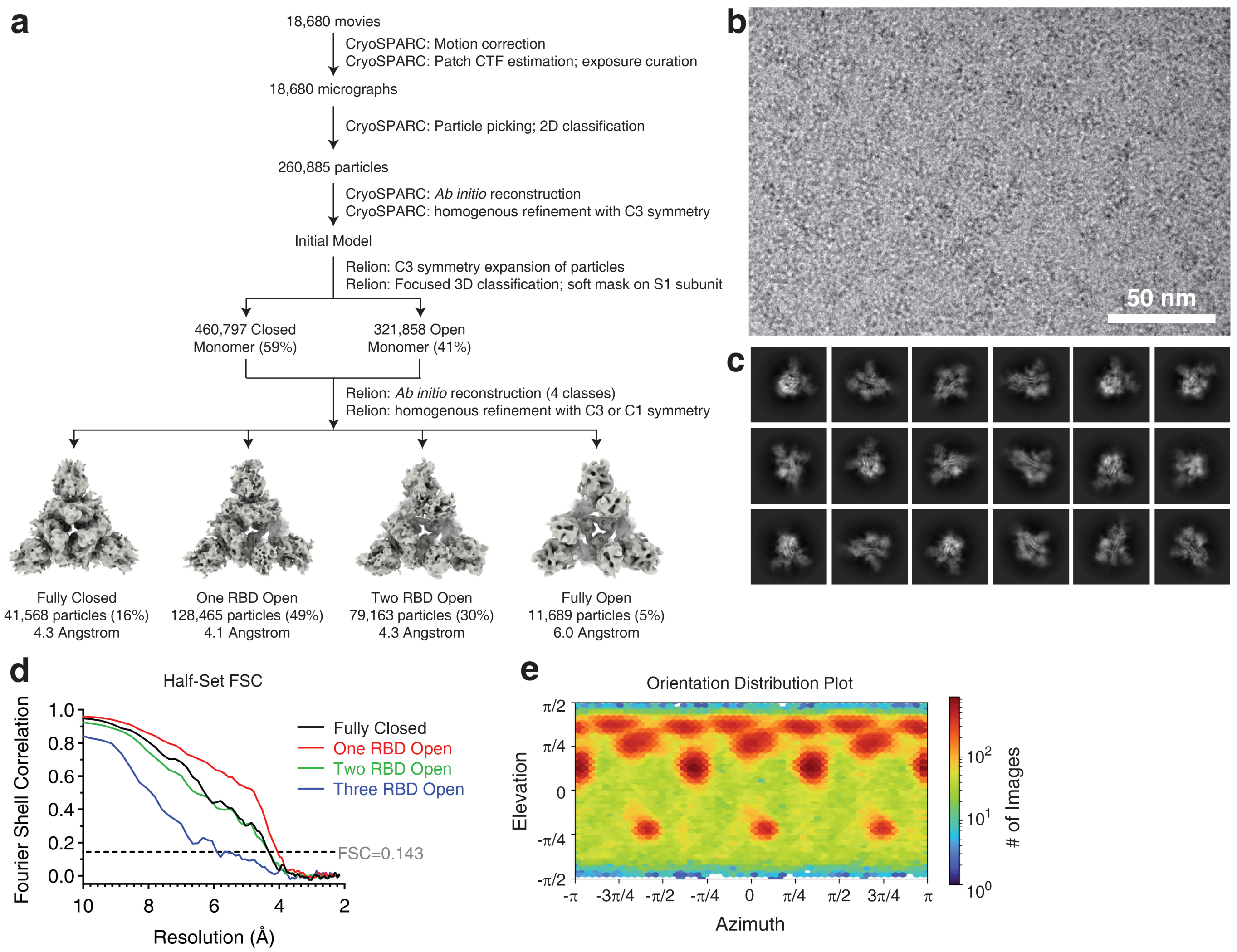

Supplementary Figure 3. Structural determination of the SARS-CoV-2 D614G Spike at physiological pH (pH 7.4) in the presence of 0.5% CHAPS. (a) Workflow for the data processing of the D614G Spike in the presence of 0.5% CHAPS. **(b)** A representative raw cryo-EM micrograph for this dataset is shown. In total, 18,680 micrographs were collected and analyzed for this dataset. **(c)** 2D clustering of extracted particles reveals identifiable features. **(d)** Half-set gold-standard Fourier shell correlation (FCS) for the four classes of Spike conformations identified in this dataset. **(e)** Orientation distribution plot for the D614G Spike in the presence of 0.5% CHAPS.

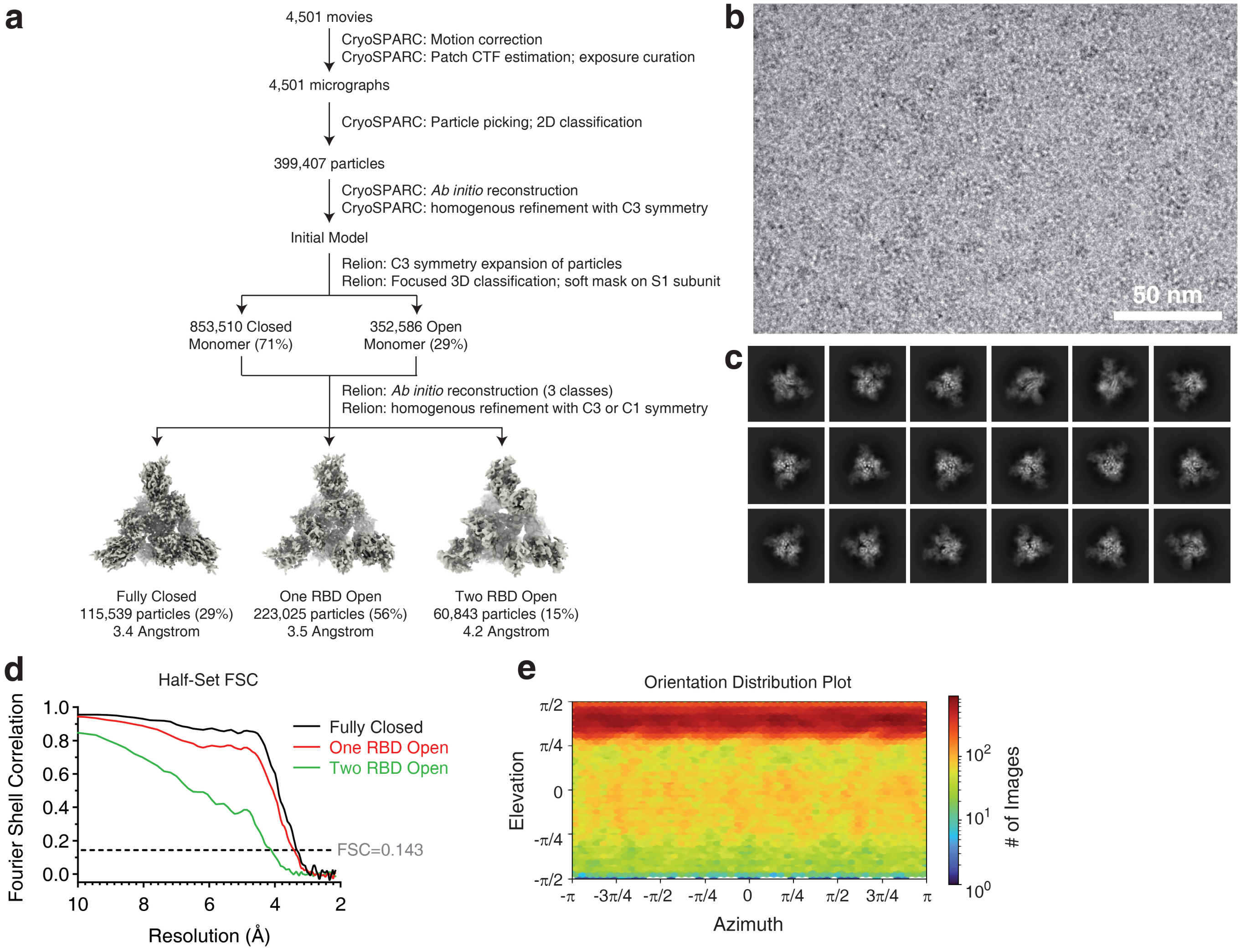

Supplementary Figure 4. Structural determination of the SARS-CoV-2 D614G Spike at physiological pH (pH 7.4) in the presence of 0.01% DDM. (a) Workflow for the data processing of the D614G Spike in the presence of 0.01% DDM. **(b)** A representative raw cryo-EM micrograph for this dataset is shown. In total, 4,501 micrographs were collected and analyzed for this dataset. **(c)** 2D clustering of extracted particles reveals identifiable features. **(d)** Half-set gold-standard Fourier shell correlation (FCS) for the three classes of Spike conformations identified in this dataset. **(e)** Orientation distribution plot for the D614G Spike in the presence of 0.01% DDM.

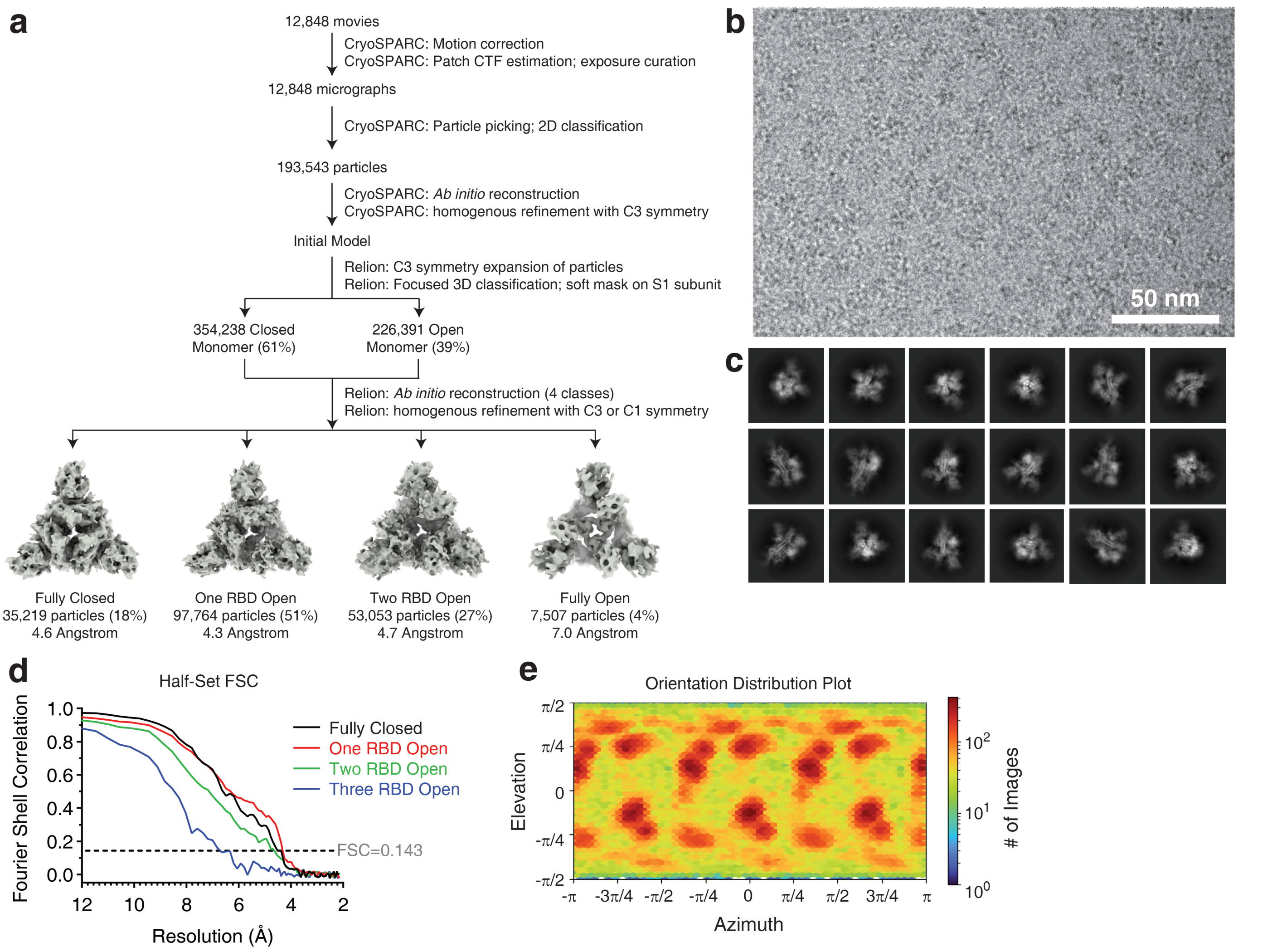

Supplementary Figure 5. Structural determination of the SARS-CoV-2 D614G Spike at physiological pH (pH 7.4) in the presence of 0.5% DDM. (a) Workflow for the data processing of the D614G Spike in the presence of 0.5% DDM. **(b)** A representative raw cryo-EM micrograph for this dataset is shown. In total, 12,848 micrographs were collected and analyzed for this dataset. **(c)** 2D clustering of extracted particles reveals identifiable features. **(d)** Half-set gold-standard Fourier shell correlation (FCS) for the four classes of Spike conformations identified in this dataset. **(e)** Orientation distribution plot for the D614G Spike in the presence of 0.5% DDM.

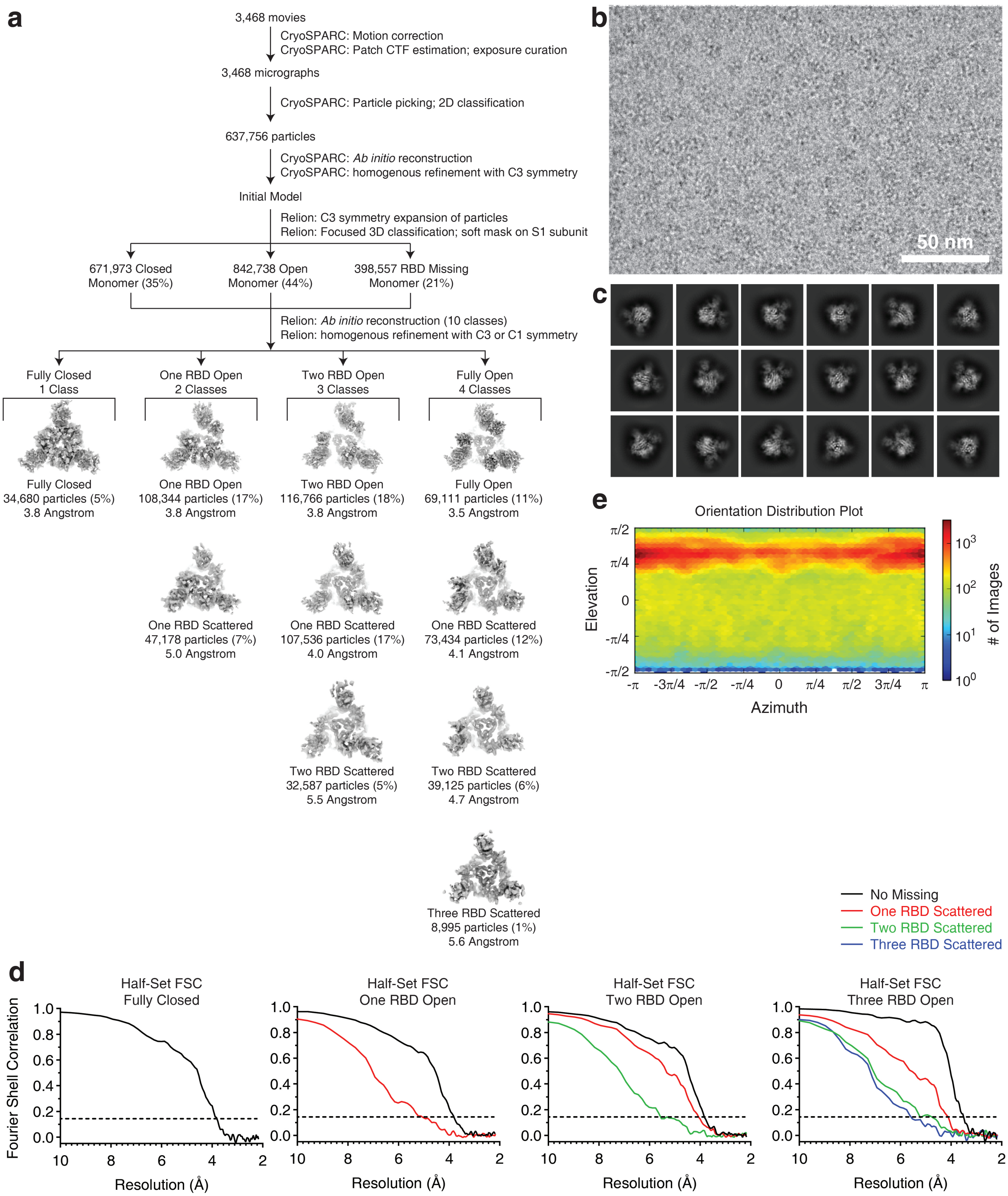

Supplementary Figure 6. Structural determination of the SARS-CoV-2 D614G Spike at acidic pH (pH 5) in the absence of detergent. (a) Workflow for the data processing of the D614G Spike in the absence of detergent. **(b)** A representative raw cryo-EM micrograph for this dataset is shown. In total, 3,468 micrographs were collected and analyzed for this dataset. **(c)** 2D clustering of extracted particles reveals identifiable features. **(d)** Half-set gold-standard Fourier shell correlation (FCS) for the ten classes of Spike conformations identified in this dataset. **(e)** Orientation distribution plot for the D614G Spike in the absence of detergent.

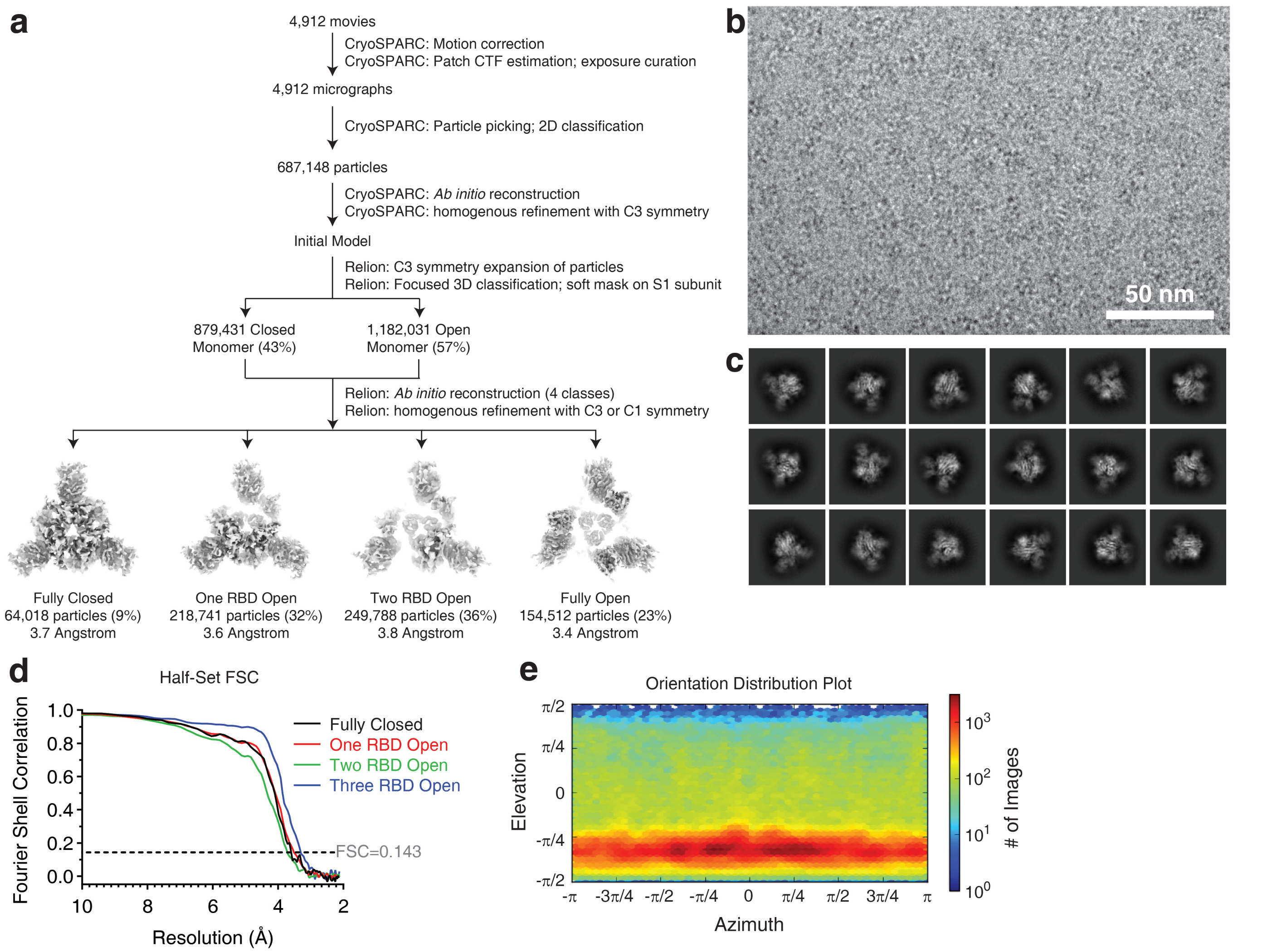

Supplementary Figure 7. Structural determination of the SARS-CoV-2 D614 Spike at acidic pH (pH 5) in the absence of detergent. (a) Workflow for the data processing of the D614 Spike in the absence of detergent. **(b)** A representative raw cryo-EM micrograph for this dataset is shown. In total, 4,912 micrographs were collected and analyzed for this dataset. **(c)** 2D clustering of extracted particles reveals identifiable features. **(d)** Half-set gold-standard Fourier shell correlation (FCS) for the four classes of Spike conformations identified in this dataset. **(e)** Orientation distribution plot for the D614 Spike in the absence of detergent.

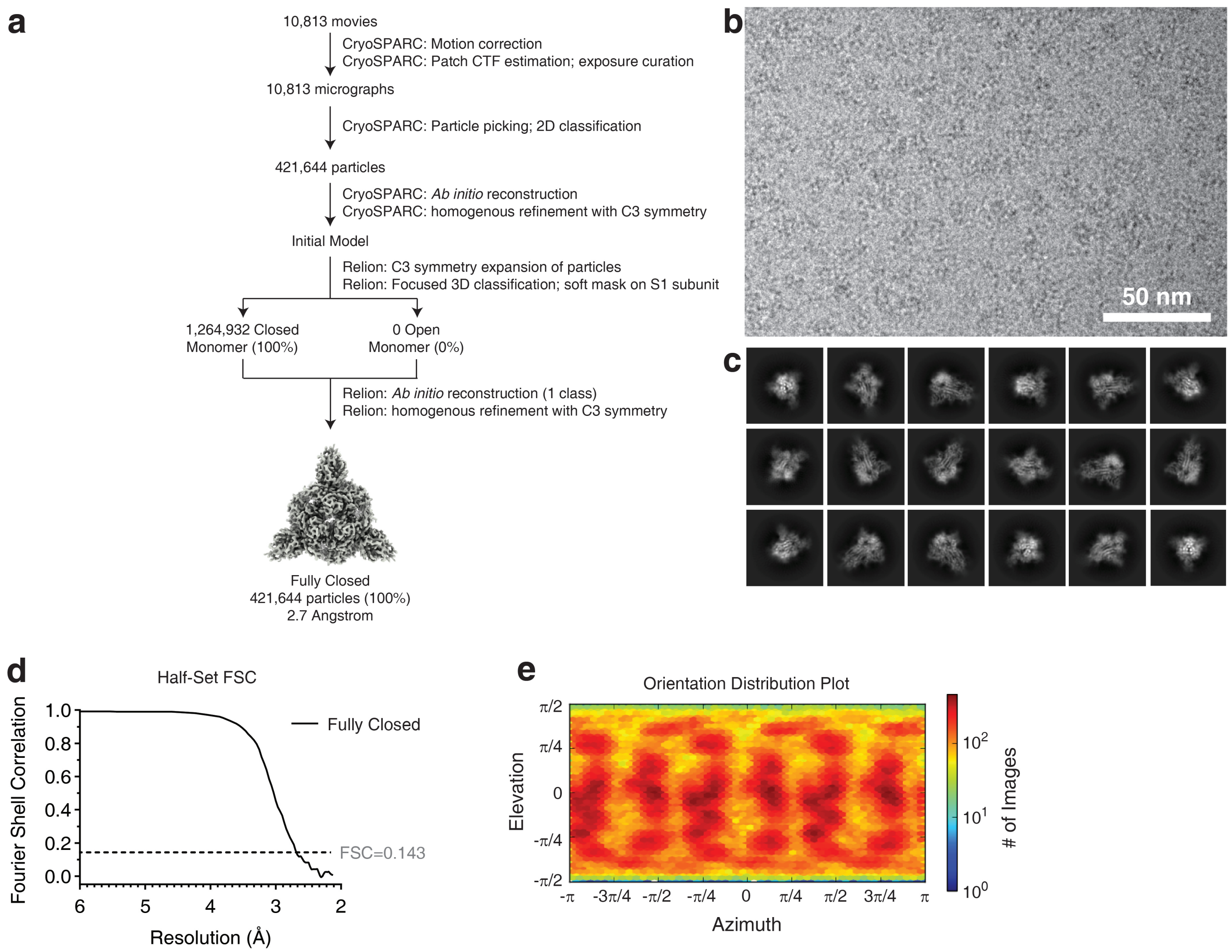

Supplementary Figure 8. Structural determination of the SARS-CoV-2 D614G Spike at acidic pH (pH 5) in the presence of 0.5% CHAPS. (a) Workflow for the data processing of the D614G Spike in the presence of 0.5% CHAPS at acidic pH. **(b)** A representative raw cryo-EM micrograph for this dataset is shown. In total, 10,813 micrographs were collected and analyzed for this dataset. **(c)** 2D clustering of extracted particles reveals identifiable features. **(d)** Half-set gold-standard Fourier shell correlation (FCS) for the fully closed conformation of the Spike protein identified in this dataset. **(e)** Orientation distribution plot for the D614G Spike in the presence of 0.5% CHAPS at acidic pH.

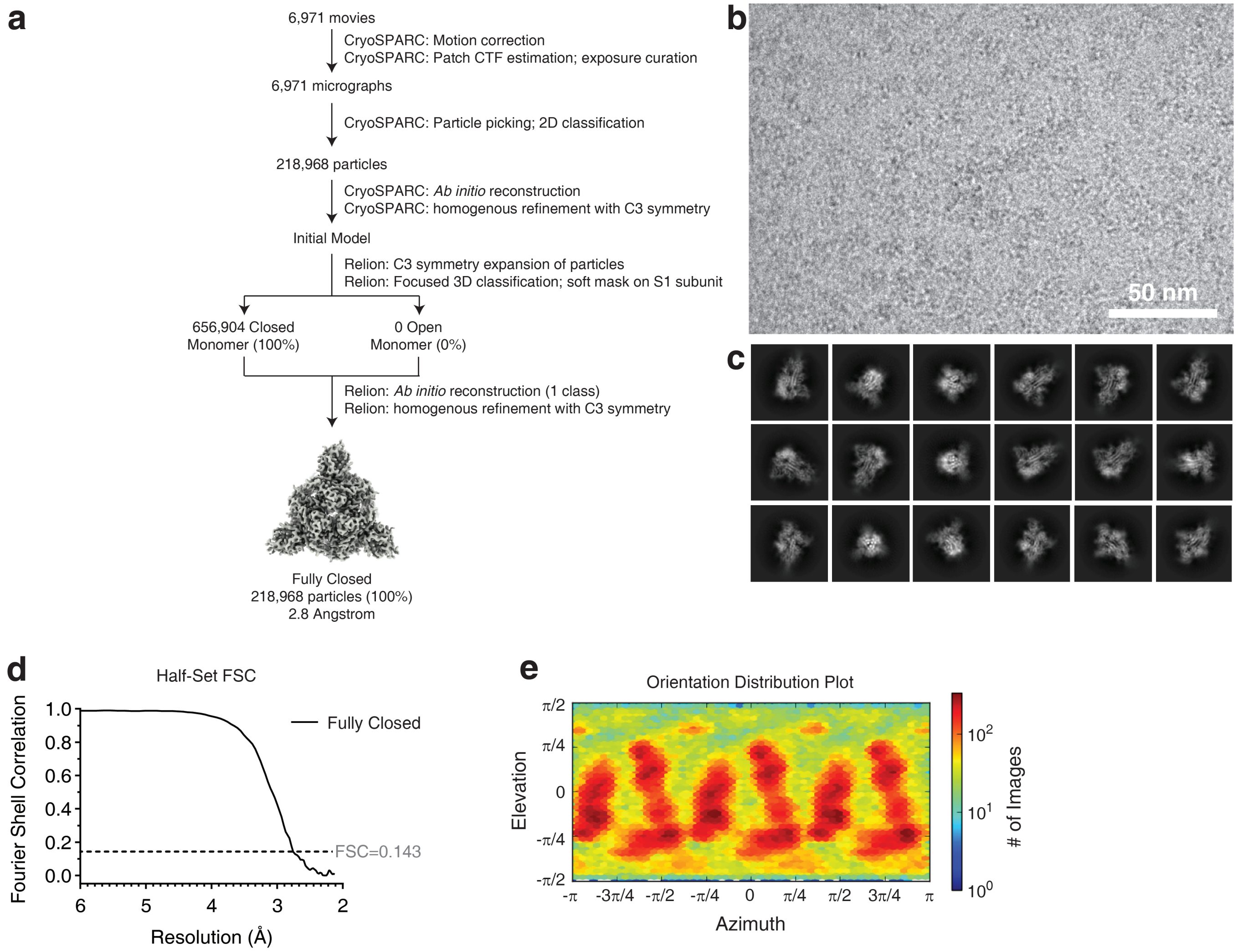

Supplementary Figure 9. Structural determination of the SARS-CoV-2 D614 Spike at acidic pH (pH 5) in the presence of 0.5% CHAPS. (a) Workflow for the data processing of the D614 Spike in the presence of 0.5% CHAPS at acidic pH. **(b)** A representative raw cryo-EM micrograph for this dataset is shown. In total, 6,971 micrographs were collected and analyzed for this dataset. **(c)** 2D clustering of extracted particles reveals identifiable features. **(d)** Half-set gold-standard Fourier shell correlation (FCS) for the fully closed conformation of the Spike protein identified in this dataset. **(e)** Orientation distribution plot for the D614 Spike in the presence of 0.5% CHAPS at acidic pH.

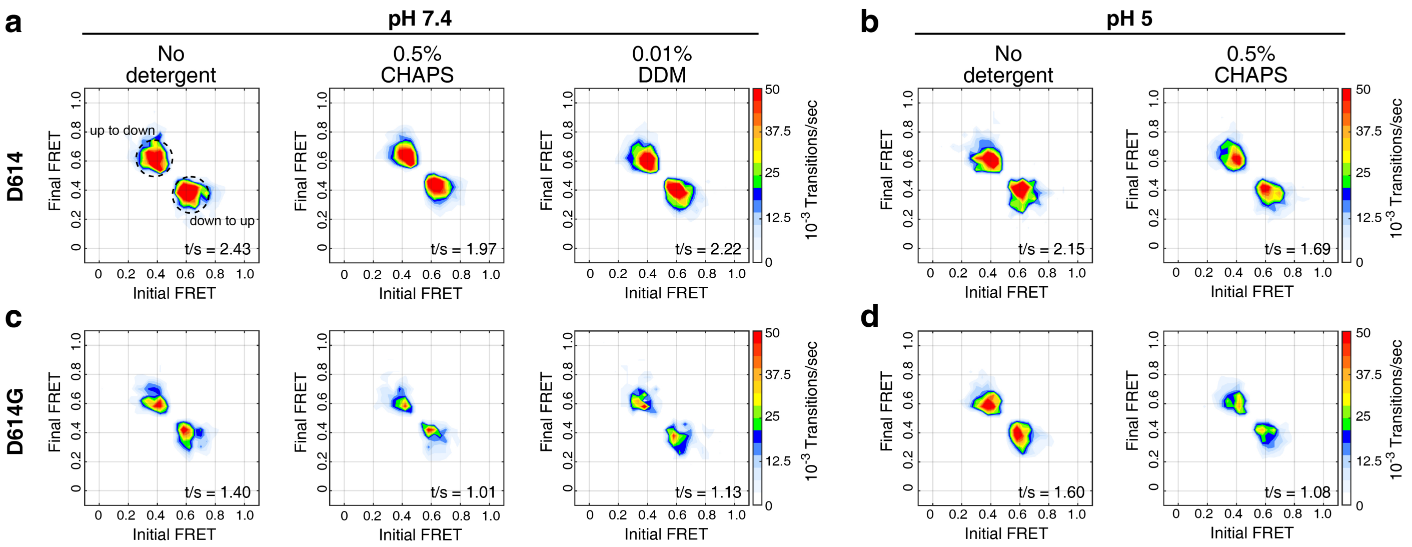

e

Variant	*pH*	*Condition*	*Low-FRET (%)*	*High-FRET (%)*	*p-value (vs. detergent-free)*	*N (# of traces)*	*t/s (Transitions/sec)*
D614	7.4	No detergent	44 ± 3	56 ± 3	-	139	2.43
D614	7.4	0.5% CHAPS	42 ± 2	58 ± 2	0.73	163	1.97
D614	7.4	0.01% DDM	43 ± 3	57 ± 3	0.95	397	2.22
D614	5.0	No detergent	55 ± 4	45 ± 4	-	97	2.15
D614	5.0	0.5% CHAPS	54 ± 2	46 ± 2	0.85	264	1.69
D614G	7.4	No detergent	60 ± 3	40 ± 3	-	142	1.40
D614G	7.4	0.5% CHAPS	59 ± 3	41 ± 3	0.96	110	1.01
D614G	7.4	0.01% DDM	56 ± 4	44 ± 4	0.64	144	1.13
D614G	5.0	No detergent	68 ± 2	32 ± 2	-	244	1.60
D614G	5.0	0.5% CHAPS	63 ± 2	37 ± 2	0.19	267	1.08

Supplementary Figure 10. Transition density plots for single-molecule FRET experiments indicate acidic pH (pH 5) and detergent do not significantly perturb the rate of transitions for SARS-CoV-2 Spike protein RBD. (a & c) Transition density plots (TDPs) for SARS-CoV-2 D614 (a) and D614G (c) Spike protein at physiological pH (pH 7.4) indicating the frequency of observed FRET transitions determined from HMM analysis. The assignment of the two transitions is shown on the upper-left TDP. **(b & d)** Transition density plots (TDPs) for SARS-CoV-2 D614 (b) and D614G (d) Spike protein at acidic pH (pH 5) indicating the frequency of observed FRET transitions determined from HMM analysis. **(e)** Summary of observed FRET transitions for all SARS-CoV-2 Spike conditions. P-values were calculated by one-way ANOVA analysis.

SUPPLEMENTARY TABLES

Supplementary Table 1. Summary of SARS-CoV-2 Spike protein cryo-EM structural studies (as of December 2022).

Spike variant	Additives	Stabilizing mutation	Furin cleavage site	Conformations observed	Reference
D614	No detergent	PP mutation (K986P, V987P)	RRAR(682-685)SGAG	Fully closed (53%), One RBD open (47%)	[1]Walls, *et al.*, 2020
D614	0.02% NaN_3	PP mutation (K986P, V987P)	RRAR(682-685)SGAG	One RBD open (100%)	[2]Wrapp, *et al.*, 2020
D614	Glycerol (4%)	PP mutation (K986P, V987P)	RRAR(682-685)GSAS	Fully closed (94%), One RBD open (6%)	[3]Xu, *et al.*, 2021
D614G	No detergent	PP mutation (K986P, V987P)	RRAR(682-685)ΔΔΔA	Fully closed (5%), One RBD open (36%), Two RBD open (39%), Three TBD open (20%)	[4]Yurkovetskiy, *et al.*, 2020
D614G	0.02% NaN_3 and 0.5% glycerol	No mutation	RRAR(682-685)GSAS	Fully closed (56%), One RBD open (44%)	[5]Gobeil, *et al.*, 2021
D614G	NP-40 (0.02%)	No mutation	No mutation	Fully closed (32%), One RBD open (68%)	[6]Zhang, *et al.*, 2021
D614G	Octyl glucoside (0.1%)	PP mutation (K986P, V987P)	RRAR(682-685)SRAS	Fully closed (13%), One RBD open (67%), Two RBD open (20%)	[7]Benton, *et al.*, 2021
Alpha (B.1.1.7)	DDM (0.02%)	No mutation	No mutation	Fully closed (16%), One RBD open (81%), Two RBD open (3%)	[8]Cai, *et al.*, 2021
Beta (B.1.351)	DDM (0.02%)	No mutation	No mutation	Fully closed (20%), One RBD open (80%)	[8]Cai, *et al.*, 2021
Beta (B.1.351)	Glycerol (4%)	PP mutation (K986P, V987P)	RRAR(682-685)GSAS	Fully closed (47%), One RBD open (53%)	[9]Wang, *et al.*, 2021
Kappa (B.1.617.1)	Glycerol (4%)	PP mutation (K986P, V987P)	RRAR(682-685)GSAS	Fully closed (51%), One RBD open (49%)	[9]Wang, *et al.*, 2021
Kappa (B.1.617.1)	DDM (0.02%)	No mutation	No mutation	Fully closed (54%), One RBD open (46%)	[10]Zhang, *et al.*, 2021
Delta (B.1.617.2)	Glycerol (4%)	PP mutation (K986P, V987P)	RRAR(682-685)GSAS	Fully closed (25%), One RBD open (75%)	[11]Wang, *et al.*, 2022
Delta (B.1.617.2)	0.02% NaN_3 and 0.5% glycerol	No mutation	RRAR(682-685)GSAS	Fully closed (38%), One RBD open (56%), Two RBD open (6%)	[12]Gobeil, *et al.*, 2022

Delta (B.1.617.2)	DDM (0.02%)	No mutation	No mutation	Fully closed (31%), One RBD open (69%)	[10]Zhang, *et al.*, 2021
Gamma (B.1.1.28)	DDM (0.02%)	No mutation	No mutation	One RBD open (100%)	[10]Zhang, *et al.*, 2021
Omicron (B1.1.529.1)	DDM (0.02%)	No mutation	No mutation	Fully closed (40%), One RBD open (60%)	[13]Zhang, *et al.*, 2022
Omicron (B1.1.529.1)	0.02% NaN_3 and 0.5% glycerol	No mutation	RRAR(682-685)GSAS	Fully closed (42%), One RBD open (35%), Two RBD open (23%)	[12]Gobeil, *et al.*, 2022
Omicron (B1.1.529.1)	CHAPSO (8 mM)	PP mutation (K986P, V987P)	No mutation	One RBD open (100%)	[14]Ye, *et al.*, 2022
Omicron (B1.1.529.1)	Glycerol (4%)	PP mutation (K986P, V987P)	RRAR(682-685)GSAS	Fully closed (61%), One RBD open (17%), Two RBD open (22%)	[15]Hong, *et al.*, 2022
Omicron (B1.1.529.1)	DDM (0.005%)	PP mutation (K986P, V987P)	RRAR(682-685)GSAS	One RBD open (100%)	[16]Cerutti, *et al.*, 2022
Omicron (B1.1.529.2)	0.02% NaN_3 and 0.5% glycerol	No mutation	RRAR(682-685)GSAS	Fully closed (79%), One RBD open (21%)	[17]Stalls, *et al.*, 2022

Supplementary Table 2. Summary of Spike conformational distribution.

Spike	pH	Detergent	Micrograph #	Particle #	Fully Closed			One RBD Open			Two RBD Open			Fully Open		
					Occ. (%)	Res. (Å)	EMDB ID	Occupancy (%)	Res. (Å)	EMDB ID	Occupancy (%)	Res. (Å)	EMDB ID	Occupancy (%)	Res. (Å)	EMDB ID
D614G	7.4	Not present	10,264	266,296	5	4.3	29525	36	3.9	29527	40	4	29528	19	3.9	29529
D614G	7.4	0.01% CHAPS	1,245	294,252	10	3.9	29428	42	3.7	29430	37	3.8	29431	11	4	29432
D614G	7.4	0.5% CHAPS	18,680	260,885	16	4.3	29434	49	4.1	29435	30	4.3	29436	5	6	29438
D614G	7.4	0.01% DDM	4,501	399,407	29	3.4	29444	56	3.5	29445	15	4.2	29446	0	—	—
D614G	7.4	0.5% DDM	12,848	193,543	18	4.6	29460	51	4.3	29461	27	4.7	29462	4	7	29463
D614G	5	Not present	3,468	637,756	6	3.8	29464	RBD observed 17<br>1 RBD scattered 7	3.8<br>5	29465<br>29466	RBD observed 18<br>1 RBD scattered 17<br>2 RBD scattered 5	3.8<br>4<br>5.5	29467<br>29468<br>29469	RBD observed 11<br>1 RBD scattered 12<br>2 RBD scattered 6<br>3 RBD scattered 1	3.5<br>4.1<br>4.7<br>5.6	29470<br>29471<br>29472<br>29473
D614	5	Not present	4,912	687,148	9	3.7	29474	32	3.6	29475	36	3.8	29476	23	3.4	29477
D614G	5	0.5% CHAPS	10,813	421,644	100	2.7	29478	0	—	—	0	—	—	0	—	—
D614	5	0.5% CHAPS	6,971	218,968	100	2.8	29479	0	—	—	0	—	—	0	—	—

SUPPLEMENTARY REFERENCES

1. Walls, A. C. *et al.* Structure, Function, and Antigenicity of the SARS-CoV-2 Spike Glycoprotein. *Cell* **181**, 281-292.e6 (2020).
2. Wrapp, D. *et al.* Cryo-EM structure of the 2019-nCoV spike in the prefusion conformation. *Science* **367**, 1260–1263 (2020).
3. Xu, C. *et al.* Conformational dynamics of SARS-CoV-2 trimeric spike glycoprotein in complex with receptor ACE2 revealed by cryo-EM. *Sci. Adv.* **7**, eabe5575 (2021).
4. Yurkovetskiy, L. *et al.* Structural and Functional Analysis of the D614G SARS-CoV-2 Spike Protein Variant. *Cell* **183**, 739-751.e8 (2020).
5. Gobeil, S. M.-C. *et al.* D614G Mutation Alters SARS-CoV-2 Spike Conformation and Enhances Protease Cleavage at the S1/S2 Junction. *Cell Rep.* **34**, 108630 (2021).
6. Zhang, J. *et al.* Structural impact on SARS-CoV-2 spike protein by D614G substitution. *Science* **372**, 525–530 (2021).
7. Benton, D. J. *et al.* The effect of the D614G substitution on the structure of the spike glycoprotein of SARS-CoV-2. *Proc. Natl. Acad. Sci.* **118**, e2022586118 (2021).
8. Cai, Y. *et al.* Structural basis for enhanced infectivity and immune evasion of SARS-CoV-2 variants. *Science* **373**, 642–648 (2021).
9. Wang, Y. *et al.* Conformational dynamics of the Beta and Kappa SARS-CoV-2 spike proteins and their complexes with ACE2 receptor revealed by cryo-EM. *Nat. Commun.* **12**, 7345 (2021).
10. Zhang, J. *et al.* Membrane fusion and immune evasion by the spike protein of SARS-CoV-2 Delta variant. *Science* **374**, 1353–1360 (2021).
11. Wang, Y. *et al.* Structural basis for SARS-CoV-2 Delta variant recognition of ACE2 receptor and broadly neutralizing antibodies. *Nat. Commun.* **13**, 871 (2022).
12. Gobeil, S. M.-C. *et al.* Structural diversity of the SARS-CoV-2 Omicron spike. *Mol. Cell* **82**, 2050-2068.e6 (2022).
13. Zhang, J. *et al.* Structural and functional impact by SARS-CoV-2 Omicron spike mutations. *Cell Rep.* **39**, 110729 (2022).
14. Ye, G., Liu, B. & Li, F. Cryo-EM structure of a SARS-CoV-2 omicron spike protein ectodomain. *Nat. Commun.* **13**, 1214 (2022).
15. Hong, Q. *et al.* Molecular basis of receptor binding and antibody neutralization of Omicron. *Nature* **604**, 546–552 (2022).
16. Cerutti, G. *et al.* Cryo-EM structure of the SARS-CoV-2 Omicron spike. *Cell Rep.* **38**, 110428 (2022).
17. Stalls, V. *et al.* Cryo-EM structures of SARS-CoV-2 Omicron BA.2 spike. *Cell Rep.* **39**, 111009 (2022).
