# Supplementary material for: Detergent modulates the conformational equilibrium of SARS-CoV-2 Spike during cryo-EM structural determination
Source: Nat Commun. 2023 May 3;14:2527. doi: 10.1038/s41467-023-38251-9 (PMC10154187; doi:10.1038/s41467-023-38251-9)
Supplement: Supplementary file 3 — Reporting Summary [file 41467_2023_38251_MOESM3_ESM.pdf]

nature portfolio

Corresponding author(s): Dr. Kuang Shen, Dr. James Munro, Dr. Natalya Dudkina

Last updated by author(s): Apr 11, 2023

Reporting Summary

Nature Portfolio wishes to improve the reproducibility of the work that we publish. This form provides structure for consistency and transparency in reporting. For further information on Nature Portfolio policies, see our Editorial Policies and the Editorial Policy Checklist.

Statistics

For all statistical analyses, confirm that the following items are present in the figure legend, table legend, main text, or Methods section.

n/a	Confirmed	
☐	☒	The exact sample size (*n*) for each experimental group/condition, given as a discrete number and unit of measurement
☐	☒	A statement on whether measurements were taken from distinct samples or whether the same sample was measured repeatedly
☐	☒	The statistical test(s) used AND whether they are one- or two-sided<br>*Only common tests should be described solely by name; describe more complex techniques in the Methods section.*
☒	☐	A description of all covariates tested
☒	☐	A description of any assumptions or corrections, such as tests of normality and adjustment for multiple comparisons
☐	☒	A full description of the statistical parameters including central tendency (e.g. means) or other basic estimates (e.g. regression coefficient) AND variation (e.g. standard deviation) or associated estimates of uncertainty (e.g. confidence intervals)
☐	☒	For null hypothesis testing, the test statistic (e.g. *F*, *t*, *r*) with confidence intervals, effect sizes, degrees of freedom and *P* value noted<br>*Give P values as exact values whenever suitable.*
☒	☐	For Bayesian analysis, information on the choice of priors and Markov chain Monte Carlo settings
☒	☐	For hierarchical and complex designs, identification of the appropriate level for tests and full reporting of outcomes
☒	☐	Estimates of effect sizes (e.g. Cohen's *d*, Pearson's *r*), indicating how they were calculated

Our web collection on statistics for biologists contains articles on many of the points above.

Software and code

Policy information about availability of computer code

Data collection	EPU 2 software was used to acquire cryo-electron microscopy movies.<br>Micromanager v2.0 was used to acquire single-molecule FRET data.
Data analysis	All single-molecule FRET data were processed and analyzed using the SPARTAN software package (https://www.scottcblanchardlab.com/software) in Matlab (Mathworks, Natick, MA).<br>All cryo-electron microscopy data processing was carried out using Relion (version 3.1) and cryoSPARC (version 3.1).

For manuscripts utilizing custom algorithms or software that are central to the research but not yet described in published literature, software must be made available to editors and reviewers. We strongly encourage code deposition in a community repository (e.g. GitHub). See the Nature Portfolio guidelines for submitting code & software for further information.

Data

Policy information about availability of data

All manuscripts must include a data availability statement. This statement should provide the following information, where applicable:

- Accession codes, unique identifiers, or web links for publicly available datasets
- A description of any restrictions on data availability
- For clinical datasets or third party data, please ensure that the statement adheres to our policy

Source data are provided with this paper. The cryo-EM density maps determined at different detergent conditions have been deposited in the Electron Microscopy

Data Bank (EMDB) with the accession codes as following: EMD-29428 [https://www.ebi.ac.uk/emdb/EMD-29428], EMD-29430 [https://www.ebi.ac.uk/emdb/EMD-29430], EMD-29431 [https://www.ebi.ac.uk/emdb/EMD-29431], EMD-29432 [https://www.ebi.ac.uk/emdb/EMD-29432], EMD-29434 [https://www.ebi.ac.uk/emdb/EMD-29434], EMD-29435 [https://www.ebi.ac.uk/emdb/EMD-29435], EMD-29436 [https://www.ebi.ac.uk/emdb/EMD-29436], EMD-29438 [https://www.ebi.ac.uk/emdb/EMD-29438], EMD-29444 [https://www.ebi.ac.uk/emdb/EMD-29444], EMD-29445 [https://www.ebi.ac.uk/emdb/EMD-29445], EMD-29446 [https://www.ebi.ac.uk/emdb/EMD-29446], EMD-29460 [https://www.ebi.ac.uk/emdb/EMD-29460], EMD-29461 [https://www.ebi.ac.uk/emdb/EMD-29461], EMD-29462 [https://www.ebi.ac.uk/emdb/EMD-29462], EMD-29463 [https://www.ebi.ac.uk/emdb/EMD-29463], EMD-29464 [https://www.ebi.ac.uk/emdb/EMD-29464], EMD-29465 [https://www.ebi.ac.uk/emdb/EMD-29465], EMD-29466 [https://www.ebi.ac.uk/emdb/EMD-29466], EMD-29467 [https://www.ebi.ac.uk/emdb/EMD-29467], EMD-29468 [https://www.ebi.ac.uk/emdb/EMD-29468], EMD-29469 [https://www.ebi.ac.uk/emdb/EMD-29469], EMD-29470 [https://www.ebi.ac.uk/emdb/EMD-29470], EMD-29471 [https://www.ebi.ac.uk/emdb/EMD-29471], EMD-29472 [https://www.ebi.ac.uk/emdb/EMD-29472], EMD-29473 [https://www.ebi.ac.uk/emdb/EMD-29473], EMD-29474 [https://www.ebi.ac.uk/emdb/EMD-29474], EMD-29475 [https://www.ebi.ac.uk/emdb/EMD-29475], EMD-29476 [https://www.ebi.ac.uk/emdb/EMD-29476], EMD-29477 [https://www.ebi.ac.uk/emdb/EMD-29477], EMD-29478 [https://www.ebi.ac.uk/emdb/EMD-29478], EMD-29479 [https://www.ebi.ac.uk/emdb/EMD-29479], EMD-29525 [https://www.ebi.ac.uk/emdb/EMD-29525], EMD-29527 [https://www.ebi.ac.uk/emdb/EMD-29527], EMD-29528 [https://www.ebi.ac.uk/emdb/EMD-29528], EMD-29529 [https://www.ebi.ac.uk/emdb/EMD-29529]. The single-molecule FRET raw data generated in this study are provided in the Source Data file.

Human research participants

Policy information about studies involving human research participants and Sex and Gender in Research.

Reporting on sex and gender	Human research participants were not included in this study, therefore reporting on sex and gender is not applicable.
Population characteristics	Human research participants were not included in this study, therefore reporting on population characteristics is not applicable.
Recruitment	Human research participants were not included in this study, therefore reporting on recruitment is not applicable.
Ethics oversight	Human research participants were not included in this study, therefore reporting on ethics oversight is not applicable.

Note that full information on the approval of the study protocol must also be provided in the manuscript.

Field-specific reporting

Please select the one below that is the best fit for your research. If you are not sure, read the appropriate sections before making your selection.

[x] Life sciences [ ] Behavioural & social sciences [ ] Ecological, evolutionary & environmental sciences

For a reference copy of the document with all sections, see nature.com/documents/nr-reporting-summary-flat.pdf

Life sciences study design

All studies must disclose on these points even when the disclosure is negative.

Sample size	For cryo-EM studies, particle numbers in the range of tens of thousands to hundreds of thousands (per dataset) is sufficient for high-resolution structures. All of our cryo-EM datasets were within this range. For smFRET measurements, we used sample sizes commensurate with similar previous studies (Diaz-Salinas, et al., eLife, 2022).
Data exclusions	Data were not excluded from analysis.
Replication	Cryo-EM data were collected on one biological sample with replicates. All single-molecule FRET measurements were performed using three technical replicates, all of which were reproducible.
Randomization	For cryo-EM, each population of particles corresponding to each dataset was randomly split into two halves which were then independently refined in RELION and cryoSPARC. The resolution of the cryo-EM maps was then assessed using Fourier shell correlation at the 0.143 gold-standard threshold. This is a standard approach to assessing cryo-EM quality and is a method to show that the resolution from each dataset is reproducible. For the smFRET measurements, randomization was not applied. This is because the behavior of individual molecules in the smFRET measurements should not display a systematic bias towards a particular conformation. Because all datasets were collected using the same smFRET experimental conditions, any bias that could be introduced would be applied equally across all datasets. Thus, randomization does not apply to this study.
Blinding	Blinding is not applicable. For cryo-EM, randomized particle assignment to half-sets followed by resolution estimates at the gold-standard cutoff were performed automatically by the cryo-EM data processing softwares described in both the manuscript and earlier in the reporting summary. Furthermore, applying blinding to the study of individual protein molecules by smFRET is not possible, nor applicable.

Reporting for specific materials, systems and methods

We require information from authors about some types of materials, experimental systems and methods used in many studies. Here, indicate whether each material, system or method listed is relevant to your study. If you are not sure if a list item applies to your research, read the appropriate section before selecting a response.

Materials & experimental systems

n/a	Involved in the study	
☒	☐	Antibodies
☐	☒	Eukaryotic cell lines
☒	☐	Palaeontology and archaeology
☒	☐	Animals and other organisms
☒	☐	Clinical data
☒	☐	Dual use research of concern

Methods

n/a	Involved in the study	
☒	☐	ChIP-seq
☒	☐	Flow cytometry
☒	☐	MRI-based neuroimaging

Eukaryotic cell lines

Policy information about cell lines and Sex and Gender in Research

Cell line source(s)	FreeStyle 293-F cells (human, ThermoFisher R79007)<br>ExpiCHO-S cells (human, Thermo Scientific A29127, Waltham, MA, USA)
Authentication	The cell lines we utilized were directly purchased from ThermoFisher with certifications from the company. They are not included on the list of known misidentified cell lines maintained by the International Cell Line Authentication Committee.
Mycoplasma contamination	Cell lines were not tested for mycoplasma contamination but no indication of contamination was observed.
Commonly misidentified lines<br>(See ICLAC register)	No commonly misidentified cell lines were used.
